# Durable complete response with chemo-immuno-radiotherapy for metastatic cervical cancer progressing under immunotherapy: a case series

**DOI:** 10.1016/j.gore.2025.101967

**Published:** 2025-10-01

**Authors:** Thomas Westerhoff, Nathalie Baudoux, Manuela Undurraga, Patrick Petignat, Intidhar Labidi-Galy, Melpomeni Kountouri

**Affiliations:** a Department of Oncology, Hôpitaux Universitaires de Genève, Genève, Switzerland; b Division of Gynecology, Department of Pediatrics and Gynecology, Hôpitaux Universitaires de Genève, Genève, Switzerland; c Faculty of Medicine, Department of Medicine and Center of Translational Research in Onco-Hematology, University of Geneva, Swiss Cancer Center Leman, Genève, Switzerland; d Department of Oncology, Division of Radiation Oncology, Hôpitaux Universitaires de Genève, Genève, Switzerland; e Department of Radiation Oncology, Hôpital fribourgeois, Fribourg, Switzerland

**Keywords:** Cervical cancer, Metastatic, Immunotherapy, Chemo-radiotherapy

## Abstract

**Objectives:**

The prognosis for metastatic cervical cancer has dramatically improved in the past years with the addition of anti-PD1/PD-L1 immunotherapy to standard platinum-based chemotherapy +/-bevacizumab. Here, we aimed to investigate the benefit from combining chemo-immuno-radiotherapy for metastatic cervical cancer patients who had oligo-metastasis progression under maintenance immunotherapy, while the other metastatic sites are in remission.

**Methods:**

We report a case series of two patients diagnosed with multisite metastatic cervical cancer (including peritoneal carcinomatosis) who, after an initial response to pembrolizumab, presented with pelvic progression while maintaining complete regression of extra-pelvic metastases. Both patients underwent radical pelvic external beam radiotherapy (EBRT) and concomitant chemotherapy with weekly cisplatin +/- brachytherapy, while pursuing immunotherapy with pembrolizumab.

**Results:**

The two patients achieved durable complete response after completing this treatment scheme and tolerated it well. Patient 1 is in complete response 30 months after the end of chemo-radiotherapy. Patient 2 is in complete response 24 months after the end of chemo-radiotherapy.

**Conclusions:**

This case series suggests that chemo-immuno-radiotherapy with EBRT may represent a salvage treatment for patients who develop secondary loco-regional resistance to immunotherapy, as long as there is remission of the metastatic sites. Given the low response rate to chemotherapy in metastatic cervical cancer patients resistant to pembrolizumab, chemo-radiotherapy while pursuing immunotherapy should be investigated in highly selected patients with oligo-metastasis progression.

## Highlights

• Successful chemo-immuno-radiotherapy in progressing metastatic cervical cancer that initially responded to immunotherapy.
• Chemo-radiotherapy was able to overcome secondary resistance to immunotherapy.
• Chemo-radiotherapy should be discussed when the progression is localized and the patient initially responded to immunotherapy.
• Both patients achieved durable and complete response.

## 1 Introduction

The prognosis of metastatic cervical cancer has significantly improved in recent years, with the addition of anti-PD1/PD-L1 immunotherapy alongside chemotherapy +/- bevacizumab. Two randomized phase III trials showed an improved overall survival benefit when adding anti-PD1/PD-L1 antibodies to platinum-based chemotherapy +/- bevacizumab (Monk et al., 2023 Dec 20, Oaknin et al., 2024 Jan). The KEYNOTE-826 phase III trial showed a 40 % survival plateau at 4 years for patients with a PD-L1 combined positive score (CPS) > 10 who received combined pembrolizumab and chemotherapy (Monk et al., 2023 Dec 20). Consistent results were obtained with atezolizumab combined with chemotherapy and bevacizumab (Oaknin et al., 2024 Jan); suggesting that adding an immune-checkpoint inhibitor (ICI) may change the natural history of the disease for a subset of metastatic cervical cancer patients, with potential cure.

Current ESGO and NCCN guidelines for the treatment of stage IVB cervical cancer recommend platinum-based chemotherapy combined with pembrolizumab for patients with a CPS score > 1, +/- bevacizumab (Cibula et al., 2023 May 1, National comprehensive cancer network. NCCN Clinical Practice Guidelines in Oncology (NCCN Guidelines®), Cervical cancer, Version 4., 2025). In case of response to systemic chemotherapy, definitive pelvic radiotherapy, in previously not irradiated patients, may be considered in the presence of pelvic residual disease [3. Recent data suggest that more intensive chemoradiotherapy (CRT) regimens with curative irradiation doses increase survival outcomes compared to classical palliative radiotherapy treatments (Escande et al., 2024). However, there is limited data on the combination of chemo-immuno-radiotherapy in metastatic patients with disease limited to the pelvis. Considering that the addition of pembrolizumab to CRT improves overall survival and is the new standard of care in locally advanced cervical cancer (LACC, stage III/IVA) (Lorusso et al., 2024), these results suggest a synergetic effect of chemo-immuno-radiotherapy in advanced cervical cancer.

This case series presents two patients with FIGO stage IVB multisite metastatic cervical cancer at diagnosis who were initially in partial response under pembrolizumab, but eventually presented progression limited to the pelvis. The patients received salvage therapy with chemoradiotherapy while pursuing immunotherapy with pembrolizumab, leading to durable complete response. Staging of cervical cancer in this case series follows the FIGO 2018 guidelines.

## 2 Case descriptions

### 2.1 Case 1

Patient 1 is a 66-year-old woman, G2 P1, who presented to the emergency room for sinus tachycardia. A thoraco-abdominal CT-scan searching for pulmonary embolism found a pelvic mass. MRI assessment revealed locoregional extension of the mass with rectum involvement associated with peritoneal carcinomatosis, pleural effusion and a suspicious splenic lesion. There were mediastinal and peritoneal lymphadenopathies. Cervical biopsy showed a mildly differentiated, HPV-associated non-keratinizing squamous cell carcinoma of the uterine cervix, with a PD-L1 CPS score of 20. Thoracentesis confirmed pleural metastatic spreading.

The patient received seven chemotherapy cycles of carboplatin/paclitaxel. She presented partial response 3 months after beginning chemotherapy. Bevacizumab was added on cycle 5 but stopped after three injections due to vaginal bleeding. The chemotherapy dose was reduced to 80 % for the last cycle due to pancytopenia. Immunotherapy was not administered during chemotherapy as it was not the standard of care at the time of diagnosis. Progression occurred in multiple sites 3 months after the end of the first-line chemotherapy: pelvis, peritoneal carcinomatosis and infra-diaphragmatic lymphadenopathies. Pembrolizumab monotherapy was initiated leading to partial response with regression of mediastinal and peritoneal disease and disappearance of the splenic lesion. The main side effect was autoimmune thyroiditis leading to hypothyroidism and requiring thyroid hormone substitution.

**Fig. 1:**
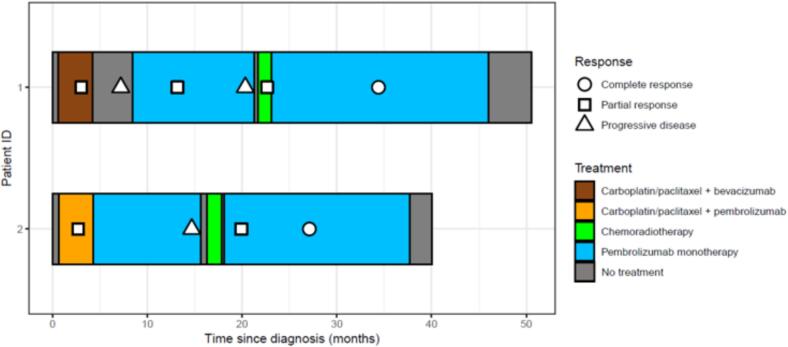
**Swimmer plot of the 2 patients.** Colors represent the treatment lines; shapes represent the response.

Twenty months after pembrolizumab initiation, the patient presented clinical progression of the cervical mass, in the form of vaginal bleeding confirmed by MRI of the pelvis. A thoraco-abdominal CT-scan showed complete response under pembrolizumab of all other metastatic lesions. Chemoradiotherapy was initiated, delivering 45 Gy in 25 fractions to the pelvic lymph nodes and the pelvis and 54 Gy in 30 fractions to the uterus and up to middle third of the vagina with external beam radiation therapy (EBRT), combined with concurrent weekly cisplatin 40 mg/m^2^ for three cycles. The patient did not undergo brachytherapy, and pursued pembrolizumab after CRT. Follow-up imaging with pelvic MRI showed partial response one month after the end of CRT. Complete response was achieved 1 year after the end of CRT and is still ongoing with 30 months follow-up, leading to stopping pembrolizumab (Fig. 1).

### 2.2 Case 2

Patient 2 is a 60-year-old woman, G4 P3, who presented to the emergency room for vaginal bleeding and pelvic pain. MRI imaging revealed a uterine mass extending to the vagina, bladder and urethra, involving parametrium on both sides, associated with left pyelocaliceal dilatation. There were retroperitoneal and pelvic lymphadenopathies and peritoneal carcinomatosis. PET-CT showed a right middle lobe lung lesion, supra- and infra-diaphragmatic lymphadenopathies, and tumor involvement of the left distal ureter. Cervical biopsy showed an HPV-associated, mildly differentiated squamous cell carcinoma, with a PD-L1 CPS score of 10.

**Fig. 2:**
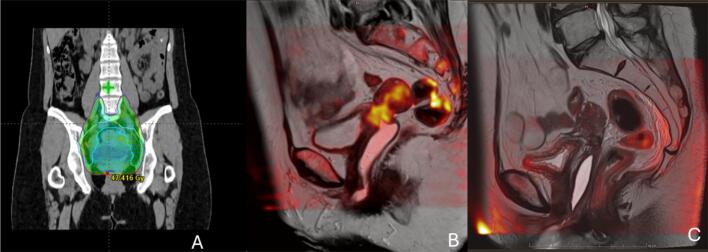
**Complete response in patient 2 with salvage CRT.** A. Radiation plan before CRT. B. Pre-treatment fusion MRI of the pelvis, showing the extent of the cervical relapse. C. Fusion MRI of the pelvis after CRT showing complete response of the cervical lesion.

The patient started chemo-immunotherapy receiving 6 cycles of carboplatin-paclitaxel and pembrolizumab every 3 weeks. The treatment led to partial response with complete regression of the cervical lesion and partial regression of thoracic and abdominal lymphadenopathies and peritoneal carcinomatosis. Maintenance pembrolizumab was pursued. After 10 months of pembrolizumab, thoraco-abdominal CT-scan and pelvic MRI revealed progression of the cervical tumor while metastatic sites had completely regressed (Fig. 2). CRT was initiated. The patient received 45 Gy in 25 fractions in the elective pelvic lymph nodes and pelvis with a simultaneous integrated boost of 55 Gy to four pelvic lymphadenopathies by EBRT combined with weekly cisplatin at 40 mg/m^2^, followed by 28 Gy of image-guided brachytherapy in 4 fractions. Maintenance pembrolizumab was reintroduced at the end of CRT. Partial response was achieved 5 months after CRT as assessed by CT-scan, with complete resolution of the cervical lesion, confirmed by pelvic MRI 2 months later (Fig. 2C). Complete response was achieved after 9 months of pembrolizumab monotherapy as assessed by PET-CT. The patient is still in complete response 24 months after the end of CRT. The treatment was well tolerated with no acute or late grade ≥ 2 gastrointestinal or urinary toxicity.

## 3 Discussion

In the current series, we report two cases of patients with stage IVB multisite metastatic cervical cancer at diagnosis, HPV-positive, PD-L1 CPS > 10 squamous cell carcinoma who achieved durable complete response after salvage chemoradiotherapy while progressing in the pelvis under immunotherapy. Both patients presented at diagnosis with multiple metastatic sites that included peritoneal carcinomatosis. Both had partial remission under pembrolizumab monotherapy for several months before progression limited to the pelvis, while extra-pelvic metastases had disappeared. The treatment regimen was well-tolerated. However, there were some differences in the treatment scheme between the patients. Patient 1 received pembrolizumab as second line, having progressed after first-line chemotherapy. Patient 2 received pembrolizumab and platinum-based chemotherapy in first line. Furthermore, Patient 2 had received a higher curative dose of radiotherapy by EBRT and image-guided brachytherapy, while Patient 1 received EBRT only.

Together, the two cases highlight the potential of combining immunotherapy and CRT to achieve a durable complete response and change the natural history of the disease in highly selected metastatic cervical cancer patients. CRT appears to be important in obtaining complete remission, considering that both patients presented secondary resistance to pembrolizumab, with progression limited to the pelvis, while extra-pelvic metastases remained in remission. The combination of CRT and anti-PD1 pembrolizumab for LACC was recently investigated in the randomized phase III trial Keynote A-18 (Lorusso et al., 2024). The study showed significant improvement of progression-free survival and overall survival particularly in stage III/IVA LACC, leading to practice changing. Our patients underwent a similar protocol, except that the treatment was administered as “salvage” therapy in the metastatic setting, with disease progressing under pembrolizumab, though limited to the pelvis.

Concerning the radiotherapy scheme, retrospective studies suggested that definitive radiotherapy leads to better overall survival than palliative chemotherapy, and an associated brachytherapy boost, delivering an ablative dose of radiotherapy, also improves survival (Wang et al., 2018). Importantly, this retrospective study included patients treated with chemotherapy alone, where median survival was 12 months. The unprecedented survival improvement obtained with chemo-immunotherapy in first line metastatic cervical cancer, with a 40 % survival plateau for patients with a CPS > 10, should encourage curative approaches in women with disease limited to the pelvis. The presented cases show that salvage CRT while pursuing pembrolizumab could achieve curative goals, in highly selected patients whose extra-pelvic metastatic disease was controlled by immunotherapy. Considering that complete response was achieved despite loco-regional progression, our cases suggest that CRT was able to overcome secondary resistance to immunotherapy. This is in line with preclinical studies that have shown that radiotherapy may induce inflammation and sensitize immunotherapy-resistant tumors to ICIs (Herrera et al., 2022 Jan, Ochoa-de-Olza et al., 2022 Jul). Given the low response rate and limited survival with second line palliative chemotherapy in metastatic cervical patients, further investigation of CRT as salvage therapeutic approach in highly selected patients is needed.

## 4 Conclusion

Durable complete response may be achieved with pelvic CRT associated to immunotherapy in highly selected metastatic cervical cancer that develop secondary resistance to anti-PD1 immunotherapy, with progression limited to the pelvis. Clinical trials are needed to confirm this therapeutic approach as an alternative to second-line chemotherapy for these patients.

## 5 Ethics

Written informed consent was obtained from the patients for publication of this case report and accompanying images.

## 6 Disclosure

ILG received consultant fees from MSD and GSK.

MK received consultant fees from MSD and GSK.

## CRediT authorship contribution statement

**Thomas Westerhoff:** Writing – original draft, Methodology, Investigation, Data curation. **Nathalie Baudoux:** Writing – review & editing, Supervision, Project administration. **Manuela Undurraga:** Writing – review & editing, Validation, Conceptualization. **Patrick Petignat:** Writing – review & editing, Project administration, Conceptualization. **Intidhar Labidi-Galy:** Writing – original draft, Validation, Supervision, Methodology, Investigation, Conceptualization. **Melpomeni Kountouri:** Writing – review & editing, Validation, Methodology, Investigation, Conceptualization.

## Declaration of Competing Interest

The authors declare that they have no known competing financial interests or personal relationships that could have appeared to influence the work reported in this paper.
